# Fear of Influenza Resurgence amid COVID-19 Pandemic: Need for Effective Flu Vaccine Still Exists

**DOI:** 10.3390/vaccines9101198

**Published:** 2021-10-18

**Authors:** Danushka K. Wijesundara, Clare Williams, Wei Sun, Andrea Marias Furuya, Yoichi Furuya

**Affiliations:** 1 The School of Chemistry and Molecular Biosciences, The Australian Institute for Bioengineering and Nanotechnology, The University of Queensland, St Lucia, QLD 4072, Australia; 2 Department of Immunology and Microbial Disease, Albany Medical College, Albany, NY 12208, USA; willicla@upstate.edu (C.W.); sunw@amc.edu (W.S.); 3 Pictor Limited, 40 Kenwyn Street, Parnell, Auckland 1052, New Zealand; a.kinga@pictordx.com

As influenza season was approaching in 2020, public health officials feared that influenza would worsen the COVID-19 situation. Co-infection of high-risk individuals with influenza virus and SARS-CoV-2 was not an unlikely scenario. Flu vaccination was highly recommended for individuals with chronic conditions that increase susceptibility to severe pulmonary infections. Against the odds, influenza virus infections remained largely quiescent during the 2020–21 winter season and the COVID-19 pandemic appeared not to be exacerbated by the influenza season and vice versa. Fortunately, public health measures, such as physical distancing and wearing of masks, that were implemented to reduce the spread of SARS-CoV-2, likely contributed to the curtailment of influenza. However, we may not be so fortunate when pre-pandemic normalcy is restored and many of the COVID-19 related restrictions are lifted. In particular, the concern is how accurately we can match the influenza vaccine strain with incoming new strains when available data on currently circulating strains is so limited due to limited appearance of influenza during the 2020–21 season. This is a reminder that broadly cross-reactive or ‘universal’ flu vaccines are still needed for prospective threats with influenza virus. This Special Issue timely presents eight articles on various aspects of the influenza vaccine with a hope that the new knowledge will aid successful vaccine development.

Despite an ongoing annual vaccination effort, substantial influenza-related mortality and morbidity occur every year worldwide. Almost 90% of influenza-associated deaths are among the elderly population in people 65 years and older [1]. One of the challenges of vaccinating the elderly population is immunosenescence. Vaccines that are effective in healthy young individuals are often inadequate for elderly adults. Paixão et al. (2021) investigated interventions that could mitigate the negative effect of aging on immune responses to influenza vaccines [2]. The group discovered that oral L-glutamine, a non-essential amino-acid, supplementation significantly increased the salivary concentration of influenza vaccine specific secretory IgA in physically active, elderly subjects. Since secretory IgA is a key effector molecule of mucosal immunity, this study demonstrates that simple intervention such as oral L-glutamine supplementation can potentially modulate vaccine-induced mucosal immunity [2].

A traditional approach for enhancing vaccine immune responses is the use of vaccine adjuvants. Isaacs et al. (2021) screened a panel of adjuvants to identify an optimal adjuvant pairing with the influenza hemagglutinin- or respiratory syncytial virus fusion protein-based subunit vaccines which were stabilized in their prefusion conformation (i.e., the conformation of these proteins found on the surface of the virion) using their proprietary ‘molecular clamp’ technology [3]. The authors found that, in mice, different adjuvants had different capacities for enhancing humoral and T cell immunity against prefusion stabilized vaccine antigens. Some adjuvants exhibited positive effects only with influenza but not with RSV antigens. Similarly, adjuvants that were able to significantly increase cellular immunity in response to RSV vaccination failed to increase the frequency of CD4+ and CD8+ IFN-γ+ cells upon influenza vaccination. They concluded that the adjuvant selection was important as the effect of the chosen adjuvant could vary greatly depending on the vaccine antigen, even if the antigens were stabilized to adopt a similar conformation (i.e., prefusion) based on an identical stabilizing domain (i.e., molecular clamp) [3].

No vaccines are risk free. A concern exists that influenza vaccines can potentially exacerbate immune-related adverse events, such as myopericarditis, in patients receiving immune checkpoint inhibitors. This possibility was explored by Gatti et al. (2020) [4] by analyzing the reported myopericarditis cases in the Vaccine Adverse Event Reporting System and VigiBase. Specifically, myopericarditis cases with influenza vaccination and immune checkpoint inhibitors as suspected triggers were retrieved for analyses. Due to the paucity of specified cases and the lack of established causation, it was concluded that the risk of myopericarditis due to the negative interaction between influenza vaccines and immune checkpoint inhibitors in cancer patients was insignificant. This report supports the overall safety of influenza vaccination for high-risk individuals, such as cancer patients.

In addition to vaccination, other non-pharmaceutical preventative measures can also positively impact the course of an infectious disease outbreak. Sun et al. (2020) analyzed febrile illness outbreak surveillance reports to identify and evaluate factors associated with influenza outbreaks in China [5]. Not surprisingly, high influenza vaccination coverage was found to prevent influenza outbreaks in schools. Further, rapid identification of febrile children and early initiation of preventative measures were also associated with reduction in the size of school-based outbreaks. These findings are consistent with what we are witnessing during the ongoing COVID-19 outbreak; rapid identification of infected individuals and early isolation can significantly retard the spread of the infectious disease.

One of the challenges in developing a universal flu vaccine was highlighted in this Special Issue; choosing an appropriate animal model that can faithfully predict vaccine efficacy in humans [6]. Most humans possess pre-existing immunity against influenza through lifetime of exposure to circulating influenza virus and/or annual flu vaccinations. Roy et al. (2020) proposed that the efficacy data from preclinical animal models often do not translate to humans because preclinical studies do not account for the pre-existing immunity and that the anti-influenza immunity can interfere with vaccine immunogenicity [6]. This hypothesis was tested and confirmed in a mouse model of sequential vaccinations/infection, which showed a negative impact of pre-existing immunity established by the inactivated influenza vaccines on subsequent intranasal live attenuated influenza vaccination [7].

The review article by McMillan et al. (2021) provides an excellent overview of the next generation influenza vaccines that are aimed to provide universal protection [8]. Sangesland et al. (2021) discussed the strategy of directing antibody responses to functionally conserved sites to confer broad protection against influenza [9]. However, these conserved regions are often immunologically silent and have reduced capacity to induce robust immune responses. The authors highlight the potential of designing structure-guided immunogens to overcome immunological subdominance [9]. This is an example of rationally designed vaccine concepts that may, in the near future, lead to a universal flu vaccine.

In summary, this issue contains eight highly relevant articles that cover various aspects of influenza vaccines. The new ideas and latest results presented in this Special Issue “Research in Immune Responses to Vaccines Antigen during Influenza A Infection” will hopefully benefit the ongoing efforts to develop a universal flu vaccine and containment of future influenza virus outbreaks.

## Data Availability

Not applicable.
